# Experiential purchases and feeling autonomous: Their implications for gratitude and ease of justification

**DOI:** 10.3389/fpsyg.2022.1033630

**Published:** 2023-01-05

**Authors:** Rogelio Puente-Diaz, Judith Cavazos-Arroyo

**Affiliations:** ^1^Faculty of Business and Economics, Universidad Anáhuac México, Anahuac, Mexico; ^2^Universidad Popular Autónoma del Estado de Puebla, Puebla, Mexico

**Keywords:** experiential purchase, material purchase, gratitude, ease of justification, autonomy support

## Abstract

In four studies, we tested the influence of type of purchase on autonomy support and the relationships between autonomy support, gratitude, and ease of justification. In each of the three studies, participants were randomly assigned to either the experiential purchase condition or the material purchase condition. In our fourth and last study, participants were assigned to an either autonomy supportive purchase condition or ordinary purchase condition. Results from study 1 showed a positive direct influence of experiential purchases on autonomy support and a direct and indirect significant relationship with gratitude. Results from study 2 with a sample of older consumers showed a positive influence of experiential purchases on autonomy support and a direct and indirect positive relationship with gratitude. In study 3, consumers who brought to mind an expensive experiential purchase reported higher autonomy support than participants who brought to mind an expensive material purchase and this experimental effect had an indirect positive relationship with gratitude and ease of justification. Last, consumers who brought to mind a purchase that truly reflected who they were reported higher levels of autonomy support than consumers who reported an ordinary purchase and this elicited autonomy had a positive relationship with gratitude. The implications of the results were discussed.

## 1. Introduction

One of the main goals of marketing is the satisfaction on consumers’ needs. Needs could be functional or abstract. Research on consumption has found a reliable affective advantage of experiential purchases over their material counterparts (Weingarten and Goodman, 2021). A recent conceptual model suggested connecting these lines of research and proposed that the advantage of experiential purchases over material ones could be explained by the satisfaction of the need for relatedness, autonomy and competence (Weingarten and Goodman, 2021). This preliminary model was developed by conducting a meta-analysis classifying the different outcomes that varied as a function of experiential versus material purchases into three categories: relatedness, autonomy, and competence (Weingarten and Goodman, 2021). As suggested by the creators of this model, the development was preliminary and tentative because they did not actually test whether the experiential advantage was only observed when the satisfaction of the need of autonomy was enhanced, or any of the other two needs. Hence, they suggested to conduct empirical studies where the satisfaction of the need for autonomy is actually assessed as a function of type of purchase. In addition, these authors suggested to: (1) increase of the scope of affective outcomes empirically tested (e.g., gratitude), (2) broaden the examination of experiential and material purchases with different characteristics (e.g., expensive purchases), and (3) examining additional marketing relevant outcomes (e.g., ease of justification). We would like to address some of the suggestions set by Weingarten and Goodman (2021). Consequently, the purpose of the present investigation is twofold. First, we test in four experiments the influence of type of purchases on the satisfaction of the need for autonomy. Second, we test how this enhanced autonomy support is related to gratitude and ease of justification. By accomplishing these objectives, this investigation seeks to make a small, incremental contribution to our understanding of the affective and consumption outcomes of type of purchase.

### 1.1. The nature of consumption

As consumers, we have the opportunity to make thousands of purchases every year to fulfill different goals and needs. The variety of goals and needs is substantial, ranging from status (Dubois and Anik, 2020), and the experience of happiness (Mogilner and Norton, 2016) to feeling ethical with one’s consumption decisions (Zane et al., 2016), among others. In addition, consumers engage in anticipated and recalled consumption. When recalling and reconstruing specific consumptions episodes, consumers have the ability to self-reflect and infer how they feel about a given purchase. As suggested in a recent model of affective outcomes and types of purchase (Weingarten and Goodman, 2021), consumers might feel that their need for relatedness, competence and autonomy is satisfied or not and need satisfaction might vary as a function of type of purchase. Specifically, this model suggests that consumers might feel more autonomous when purchasing experiences as opposed to material objects with different, seemingly more positive, affective and consumption consequences (Weingarten and Goodman, 2021).

### 1.2. The nature of autonomy and its potential interaction with purchases

The core concept of our article is autonomy. As suggested by Self-Determination Theory (SDT), autonomy represents one of the three basic human needs, along competence and relatedness, that if satisfied, leads to positive affective and behavioral outcomes (Ryan and Deci, 2000). The concept of autonomy has received increased attention and development from philosophical and psychological perspectives (Ryan and Deci, 2018). Specifically, autonomy refers to feeling as the origin of one’s actions and to feeling that one’s choices are consistent with core values of the self (Ryan and Deci, 2018). For marketing and consumer behavior, the conceptualization of autonomy as feeling that one’s choices are consistent with core values of the self might have more applicability, given that purchases represent one type of choice that consumers make.

Self-reflection, including the process of recalling and reconstruing a given purchase, allows consumers to transcend their current experiences and give value to objects, aims and life events, including purchases (Ryan and Deci, 2018). As suggested by existential and consumer behavior scholars, there is evidence that consumers assign value, beyond their functionality, to material objects (Belk, 1988; Routledge and Vess, 2019) and experiences (Zauberman et al., 2009). For example, consumers perceive certain objects and products as reflecting their identities (Wheeler and Bechler, 2021), as objects of attachments (Richins and Chaplin, 2021), and as reminders of special others in their lives (van den Hoven et al., 2021). Similarly, perceptions and reconstructions are elicited by recalling and reconstruing ordinary and extraordinary experiences (Zauberman et al., 2009; Bhattacharjee and Mogilner, 2014). Consequently, when consumers are asked to think and reflect on their purchases, they confer value to these purchases, which could be experiential and material (Fransen et al., 2019). Yet, we propose an experiential advantage over material purchases. Specifically, we posit that when thinking and reflecting on experiential purchases, consumers would feel that these purchases are more consistent with their core values, experiencing higher autonomy than when thinking and reflection on material purchases. If these propositions are correct, consumers would obtain positive affective and consumption outcomes when asked to think and reconstruct experiential purchases. After having stated the nature of consumption and autonomy, we turn our attention to reviewing that relevant literature that would justify the idea that experiential purchases are more consistent with the core values of the self and the relevant literature on the positive outcomes coming from this consistency.

### 1.3. Experiential versus material purchases

We review the relevant literature to justify our proposition that recalling experiential purchases should lead consumers to feel more autonomous than recalling their material counterparts because experiential purchases are more aligned with the true self. One study showed that experiential purchases were construed as more meaningful moments in consumer’s lives than their material counterparts (Puente-Díaz and Cavazos-Arroyo, 2021a). Similarly, experiential gifts were also construed as more meaningful moments in consumers’ lives than material gifts (Puente-Díaz and Cavazos-Arroyo, 2021b). In addition, one study showed that when telling their life stories, consumers were more likely to include experiential than material purchases in their narratives (Carter and Gilovich, 2012). Assuming that when telling life stories, one is more likely to include momentous events in which values important to the self were endorsed (Pillemer, 1998), then the inclusion of more experiential than material purchases represents evidence for the autonomy support advantage of experiential purchases. Similarly, another study showed that when consumers wanted to express their true self, they were more likely to choose experiential than material purchases (Bronner and de Hoog, 2018). Regarding the socialization of how consumers are, one study showed that experiential purchases led to more identity expression opportunities (Guevarra and Howell, 2015). Similarly, another study showed that experiences generated higher levels of word of mouth (Gallo et al., 2019), had greater conversational value (Bastos and Brucks, 2017), and were more likely to be posted in social media (Duan and Dholakia, 2018), indicating indirectly that experiences might be more consistent with the core values of the self.

From this focused review of the literature, there seems to be enough evidence to directly test the influence of experiential purchases on autonomy support. Thus far, the evidence regarding the influence of experiential purchases on relatedness support is more robust than the evidence regarding autonomy support (Weingarten and Goodman, 2021). The empirical studies reviewed showed that experiences might be more autonomy supportive, but to our knowledge, limited attention has been given to this proposition. For example, one study included an assessment of autonomy support, yet the results did not support the idea that experiential purchases lead to higher levels of autonomy support (Howell and Hill, 2009). Consequently, we seek to address this limitation by positing and testing the following hypothesis:

*H1*: Remembering an experiential purchase would lead to higher levels of autonomy support than remembering a material purchase.

As shown in previous investigations, types of purchases have affective and direct and indirect consumption consequences (Gilovich and Gallo, 2020). To broaden the scope of the current empirical literature, we focus on two important, yet overlooked outcomes: Gratitude and ease of justification.

### 1.4. Gratitude

Gratitude is a social and moral emotion, often correlated with important, positive outcomes (Ahrens and Forbes, 2014). Recently, business scholars have made calls to increase our understanding of gratitude in consumer behavior (Lee et al., 2022). Gratitude scholars distinguish between the emotion of feeling grateful to a person and feeling grateful for things in general, for the good things in life (Lambert et al., 2009). For this investigation, we would focus on feelings grateful for the good things in life, which might include the purchase of experiences and material objects. We posit that consumers would, in general, feel grateful for the purchases they are able to make, but they would experience greater feelings of gratitude for experiential purchases. The advantage of experiential over material purchase is because they represent purchases more consistent with consumers’ true values. Thus far, one investigation found a positive effect of experiential purchases on gratitude (Walker et al., 2016). However, this study did not directly assess the role of autonomy support. In addition, another investigation found that marketing campaigns focused on gratitude led consumers to make fewer materialistic decisions (Lee et al., 2022). Yet, this investigation did not examine type of purchase as an elicitor of gratitude. We suggest that the positive influence of experiential purchases on gratitude is indirect, by increasing feelings of autonomy. We posit and test the following hypothesis:

*H2*: Remembering an experiential purchase would be positively and indirectly correlated with greater gratitude by increasing feelings of autonomy.

### 1.5. Ease of justification

As documented extensively, consumers construe preferences and judgments on the spot and this construal can be done in prospect and in retrospect (Lichtenstein and Slovic, 2000). Consumers evaluate how they feel about a given purchase and try to avoid feeling regret (Bui et al., 2011), among other things. We suggest that a positive outcome coming from experiential purchases and from enhanced feelings of autonomy is that consumers would find reasons to justify their purchases with greater ease than when justifying material purchases (Simonson, 1989). This ease of justification would play a more important when consumers buy expensive experiential or material purchases. We seek to follow another recommendation from a recent meta-analysis (Weingarten and Goodman, 2021) to broaden the scope of the examination of consumption outcomes by assessing ease of justification. We conceptualize ease of justification as the sensations that: (1) it is easy to justify a purchase, (2) it is money well-spent, (3) consumers feel well about their purchase, and (4) there is little guilt and regret for the money spent. We test the following hypothesis:

*H3*: Remembering an experiential purchase would be positively and indirectly correlated with greater ease of justification by increasing feelings of autonomy.

**Figure 1:**
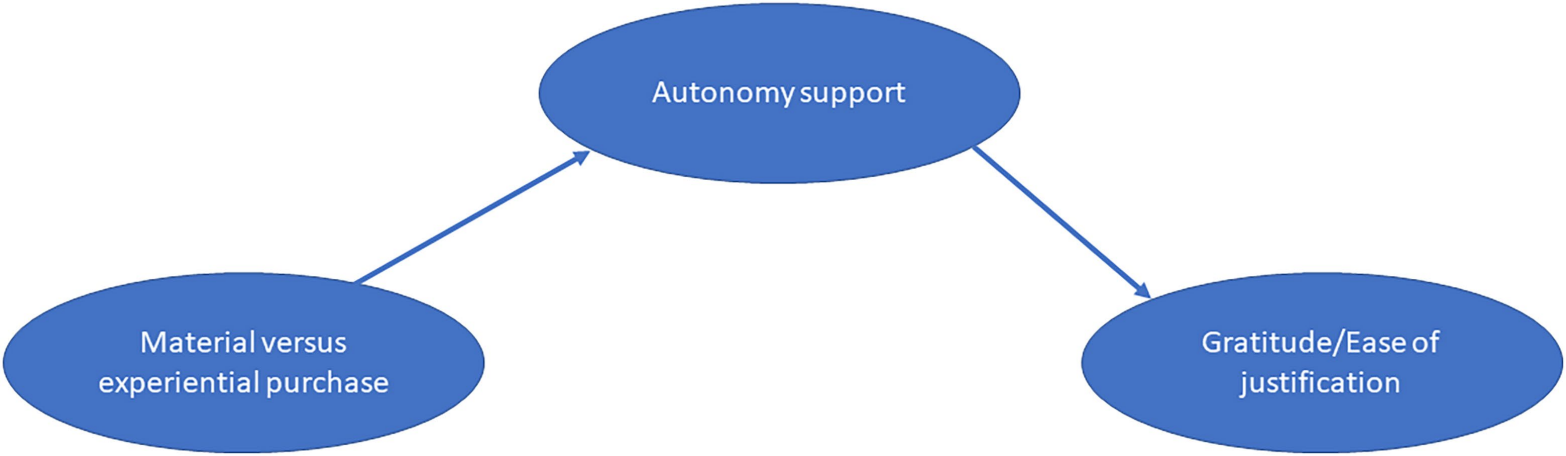
Conceptual model.

In sum, the purpose of the present investigation is twofold. First, we test in four experiments the influence of type of purchase on autonomy support. Second, we assess how these enhanced feelings of autonomy correlated with gratitude (in four studies) and ease of justification (in one study; See Figure 1 for conceptual model). Achieving these two objectives would allow us to make a small, incremental contribution to our understanding of types of purchases and their affective and consumption outcomes.

## 2. Overview of studies

In order to test our hypotheses, we followed a multi-study strategy. We first conducted an experiment in which type of purchase was experimentally manipulated and examined its direct influence on autonomy support and indirect influence on gratitude. In experimental two, we conducted a conceptual replication with an older, sample of adults to increase the generalizability of our findings. In experiment three, we again manipulated type of purchase and requested participants to bring to mind an expensive experiential or material purchase and examined its effects on gratitude and ease of justification. Last, in study four, we manipulated the proposed mediator and examined its influence on autonomy support and gratitude. We used the results from a recent meta-analysis to calculate the sample size needed for our study (Weingarten and Goodman, 2021). We conducted a power analysis *via* G*Power (Faul et al., 2007) to determine the number of participants needed for a power of 0.95 with an effect size of 0.26^1^ and two groups. Results showed that we needed a sample size of at least 196 participants.

## 3. Method of study 1

### 3.1. Participants

Participants were 412 (62% women and 38% men; 82% were in the 18–25 age range) college students from two universities in Mexico.

### 3.2. Procedure

Participants were randomly assigned to one of the two conditions: experiential or material purchase condition. Participants read the following instructions taken from previous studies (e.g., Mann and Gilovich, 2016; Puente-Díaz and Cavazos-Arroyo, 2021a):

Experiential condition: For this activity, we would like you to think about an experiential purchase that you have made with a price of around 2000 pesos (100 US dollars). An experiential purchase involves spending money with the primary intention of acquiring an event or a series of events that you have had and lived. Please describe in the space provided below such purchase.

Material condition: For this activity, we would like you to think about material purchase that you have made with a price of around 2,000 pesos (100 US dollars). A material purchase involves spending money with the primary intention of acquiring a material possession-a material product that you obtain and keep in your possession. Please describe in the space provided below such purchase. After being randomly assigned to one of two experimental conditions, participants completed a set of questionnaires, including the following measures.

### 3.3. Measures

Autonomy support and gratitude. We assessed all items by using the following prompt: Remembering this experiential (material) purchase makes me feel? In a scale from “I do not feel like that at all” (1) to “I completely feel like that” (10). The four items for autonomy support were taken from the Basic Psychological Need Satisfaction and Frustration Scale (BPNSFS; Chen et al., 2015). Sample items were: “I feel a sense of choice and freedom in the things I undertake” and “I feel that my decisions reflect what I really want.” The scores showed acceptable levels of internal consistency (*α* = 0.85). The items for gratitude were: grateful, thankful, and appreciative (*α* = 0.75), taken from the relevant literature (Frias et al., 2011). Participants were also asked to report an estimate of how much money was spent in their purchase, which did not have a significant influence on the results.

## 4. Results

### 4.1. Mediation model

In order to test for our mediation model, we followed the guidelines set by Hayes (2018). A bootstrap test (PROCESS, model 4, Hayes, 2018) showed, there was a significant effect of the experimental condition on autonomy support, *b* = 0.31, *p* = 0.007, *δ* = 0.26. The relationship between autonomy support and gratitude, while controlling for the experimental condition, was also significant, *b* = 0.61, *p* < 0.001. The influence of the experimental condition on gratitude was significant, *b* = 1.54, *p* < 0.001. Last, the indirect effect of the experimental condition on gratitude was significant, 0.19, CI = 0.05, 0.34. Hence, our results showed that bringing to mind an experiential purchase led directly to greater gratitude and indirectly by increasing feelings of autonomy support.

## 5. Brief discussion

Our results provided initial evidence for the positive influence of experiential purchases on autonomy support and the positive relationship between autonomy support and gratitude, supporting hypothesis one and two. Our results complemented the findings from a recent meta-analysis (Weingarten and Goodman, 2021) in which relatedness support was identified as an important mechanism for the positive influence of experiential purchases on happiness. One limitation of our study is that we used college students as our participants. Study 2 addressed this limitation by conducting an exact replication with an older sample to increase the generalizability of the results.

## 6. Method of study 2

### 6.1. Participants

Participants were 219 (68% women and 32% men; 59% were 36 and older) consumers from Mexico who responded to an invitation in social media to participate in this study. 35 participants failed the attention check question. Our final sample was 184 participants.

### 6.2. Procedure and measures

We used the same experimental procedure and measures as in study 1, with the exception of an additional attention check question in which participants were given the instruction to choose the number 6 as their answer. Attention check questions seek to guard the quality of online answers to questionnaires without affecting negatively the validity of scores (Kung et al., 2018).

## 7. Results

### 7.1. Mediation model

We followed the guidelines set by Hayes (2018) as in study 1. A bootstrap test (PROCESS, model 4, Hayes, 2018) showed, there was a significant effect of the experimental condition on autonomy support, *b* = 0.96, *p* < 0.001, *δ* = 0.61. The relationship between autonomy support and gratitude, while controlling for the experimental condition, was also significant, *b* = 0.70, *p* < 0.001. The influence of the experimental condition on gratitude was significant, *b* = 0.77, *p* = 0.005. Last, the indirect effect of the experimental condition on gratitude was significant, 0.68, CI = 0.33, 1.06. Hence, our results showed that bringing to mind an experiential purchase led directly to greater gratitude and indirectly by increasing feelings of autonomy support.

## 8. Brief discussion

Our results provided additional support for the positive influence of experiential purchases on autonomy support and the positive relationship between autonomy support and gratitude, supporting hypothesis one and two. In addition, we were able to replicate our results with an older population, increasing the generalizability of our findings. It was worth mentioning that the effect size of the experimental manipulation was larger than the one found in study 1. Our next study wanted to continue examining the implications of type of purchase by asking participants to bring to mind an expensive experiential or material purchase. In addition, we added ease of justification as an additional outcome of type of purchase. Specifically, we wanted to assess ease of justification for experiential and material purchases in the context of expensive purchases. By examining expensive purchases and ease of justification, we answered a recent call to broaden the scope of the types of purchases investigated and to include additional consumption outcomes (Weingarten and Goodman, 2021).

## 9. Method of study 3

### 9.1. Participants

Participants were 407 (64% women and 36% men; 85% were in the age bracket of 18–25) college students from two universities in Mexico. 31 participants failed the attention check question. Our final sample was 376 participants.

### 9.2. Procedure and measures

We used the same experimental procedure as in study 1 and 2 with the additional request of bringing to mind the most expensive experiential or material purchase participants had ever made. The mean price was $74,630 Mexican Pesos (around $3,500 USA dollars). Price did not have a significant effect on the results. We used the same autonomy support (*α* = 0.84) and gratitude questions (*α* = 0.55) and the same prompt as study 1 and 2, but added five questions to assess ease of justification of the purchase made. These five items written by reading the relevant literature were: (1) with the sensation that it is easy to justify why I made that purchase, (2) with the sensation that it was money well-spent, (3) with the sensation that I made a good purchase, (4) Without feelings of guilt for the money spent, and (5) Without any regret for the money spent. We used the same scale as study 1 and 2, “I do not feel like that at all” (1) to “I completely feel like that” (10). The scores showed acceptable levels of internal consistency (*α* = 0.81). We used the same attention check question in which participants were given the instruction to choose the number 6 as their answer.

Given that our ease of justification measure was new, we conducted a Confirmatory Factor Analysis to assess the measurement properties of the scores. Results model showed an acceptable model fit *χ*^2^ = 13.03, *p* = 0.011 (df = 4), RMSEA = 0.07, CFI = 0.99 and TLI = 0.99. Examination of the factor loadings showed that they were all significant and in the expected direction (ranging from 0.64 to 0.92). Consequently, the score of ease of justification had acceptable properties to be included as an additional dependent variable.

## 10. Results

### 10.1. Mediation model

We again followed the guidelines set by Hayes (2018) to test two mediation models, one for gratitude and one for ease of justification. A bootstrap test (PROCESS, model 4, Hayes, 2018) for gratitude showed, there was a significant effect of the experimental condition on autonomy support, *b* = 0.79, *p* < 0.001, δ = 0.60. The relationship between autonomy support and gratitude, while controlling for the experimental condition, was also significant, *b* = 0.52, *p* < 0.001. The influence of the experimental condition on gratitude was significant, *b* = 0.56, *p* < 0.001. Last, the indirect effect of the experimental condition on gratitude was significant, 0.41, CI = 0.24, 0.61.

Results for ease of justification showed a significant effect of the experimental condition on autonomy support, *b* = 0.79, *p* < 0.001. The relationship between autonomy support and ease of justification, while controlling for the experimental condition, was also significant, *b* = 0.67, *p* < 0.001. The influence of the experimental condition on ease of justification was not significant, *b* = 0.11, *p* = 0.38. Last, the indirect effect of the experimental condition on ease of justification was significant, 0.53, CI = 0.32, 0.77. From both sets of analyses, we could conclude that expensive experiential purchases had a significant, indirect effect on gratitude and ease of justification by increasing feelings of autonomy.

## 11. Brief discussion

Our results supported again the idea that experiential purchases lead to greater feelings of autonomy which then have positive relationships with gratitude and ease of justification, supporting hypothesis one, two, and three. Our experimental manipulation included the instruction of bringing to mind the most expensive experiential or material purchase, increasing the generalizability of the results from study 1 and 2. In addition, our results extended the implications of experiential purchases by assessing ease of justification as an important consumer related outcome. One limitation of the first three experiments is that we measured instead of manipulating the proposed mediator, autonomy support. As suggested by methodology scholars (Spencer et al., 2005), this represents a limitation. Consequently, in the next study, we manipulated autonomy and measured autonomy support and gratitude.

## 12. Method of study 4

### 12.1. Participants

Participants were 238 (73% women and 27% men; 78% were 35 years of age and younger) college students from two universities in Mexico. 20 participants failed the attention check question. Our final sample was 218 participants.

### 12.2. Procedure and measures

Participants were randomly assigned to one of the two conditions: autonomy supportive or ordinary purchase condition:

Autonomy supportive purchase: We, as consumers, make thousands of purchases. We would like you to think and describe a purchase that reflects or reflected who you are as a person, a purchase consistent with your values and with who you are. The price of the purchase should be around $2,000 pesos. Please describe your purchase in the space below.

Ordinary purchase: We, as consumers, make thousands of purchases. We would like you to think and describe a purchase that is or was ordinary and common for you, a routine purchase. The price of the purchase should be around $2,000 pesos. Please describe your purchase in the space below. The mean price for the reported purchases was $3,726 Mexican pesos, which was significantly smaller than the mean reported in study 3, $74,630 Mexican pesos, showing that participants followed the instructions. Price did not have a significant effect on the results.

Given the justified criticism about the underreporting of the psychometric properties of the scores coming from the scales used in experiments (Hussey and Hughes, 2020), we conducted a Confirmatory Factor Analysis to model the dimensionality of our gratitude and autonomy support measures combining the participants from all four studies (*N* > 1,200). Results for the measurement model showed an acceptable model fit *χ*^2^ = 100.47, *p* < 0.001 (df = 12), RMSEA = 0.076, CFI = 0.99, and TLI = 0.98. Examination of the factor loadings showed that they were all significant and in the expected direction (ranging from 0.51 to 0.90). The H coefficients for the scores for gratitude and autonomy support had acceptable values, 0.75 and 0.91, respectively. The bivariate latent correlation was 0.48. Hence, the results showed that the scores of gratitude and autonomy had acceptable psychometric properties.

## 13. Results

### 13.1. Mediation model

**Figure 2:**
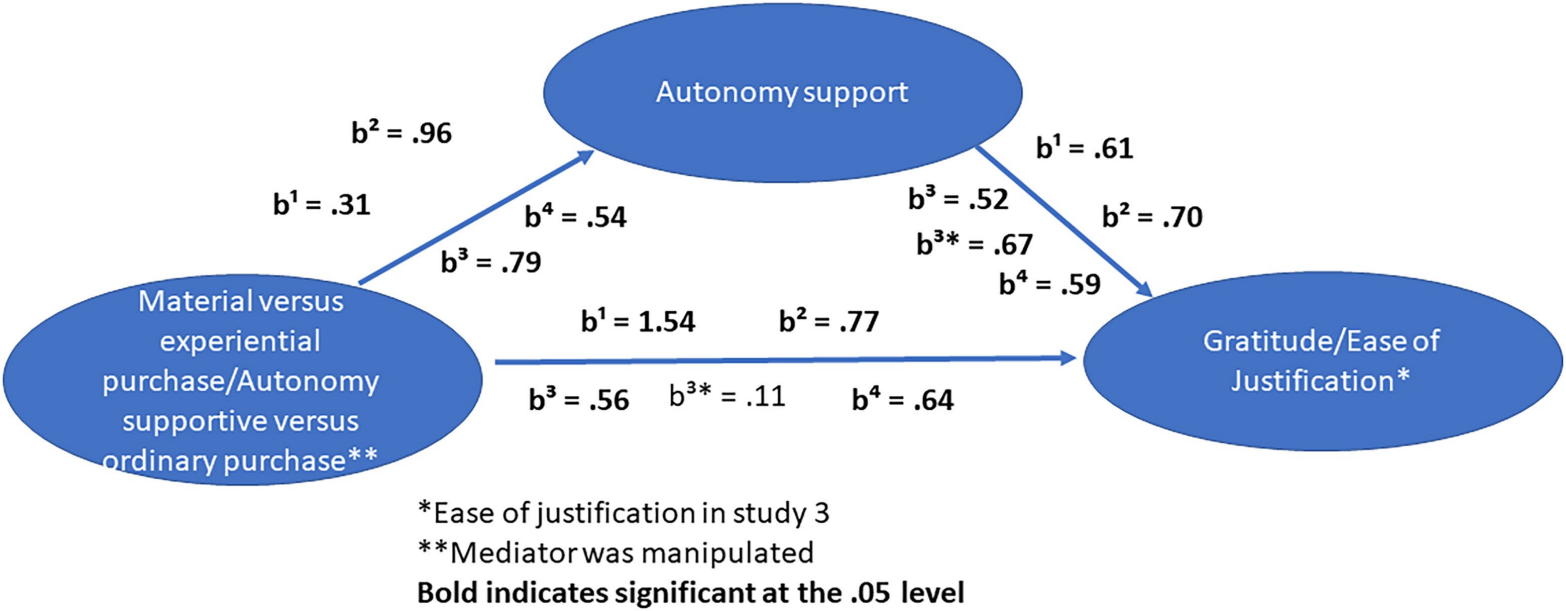
Summary of results.

We followed the guidelines set by Hayes (2018) as in study 1, 2, and 3. A bootstrap test (PROCESS, model 4, Hayes, 2018) showed, there was a significant effect of the experimental condition on autonomy support, *b* = 0.54, *p* = 0.01, *δ* = 0.38. The relationship between autonomy support and gratitude, while controlling for the experimental condition, was also significant, *b* = 0.59, *p* < 0.001. The influence of the experimental condition was significant, *b* = 0.64, *p* = 0.003. Last, the indirect effect of the experimental condition on gratitude was significant, 0.32, CI = 0.09, 0.59. Hence, our results showed that bringing to mind a purchase consistent with participants’ values led to greater autonomy support, which then had a positive relationship with gratitude (see Table 1 for descriptive statistics of all four experiments and Figure 2 for summary of results).

**Table 1:** Descriptive statistics for all four experiments.

	*M*	SD	δ	*M*	SD
	Experiential purchase			Material purchase	
Study 1: Autonomy support	9.10	1.04	0.26	8.79	1.31
Gratitude	8.49	1.66	0.88	6.75	2.26
Study 2: Autonomy support	9.10	1.16	0.61	8.14	1.91
Gratitude	7.86	1.84	0.70	6.41	2.29
	Expensive experiential purchase			Expensive material purchase	
Study 3: Autonomy support	9.20	1.02	0.60	8.41	1.55
Gratitude	8.69	1.33	0.60	7.71	1.87
Ease of Justification	9.10	1.21	0.44	8.46	1.66
	Autonomy supportive purchase			Ordinary purchase	
Study 4: Autonomy support	8.95	1.16	0.38	8.41	1.64
Gratitude	8.32	1.48	0.57	7.36	1.85

## 14. Brief discussion

Manipulating the proposed mediator, autonomy support, led participants to greater feelings of autonomy, which then had a positive relationship with gratitude. This last experiment increased our confidence about the role of autonomy support in the gratitude advantage of experiential over material purchases. The implications of our results were discussed.

## 15. General discussion

Across four studies, we found support for the propositions that experiential purchases led to greater autonomy support, which then had positive relationships with gratitude and ease of justification. Results were consistent with a sample of college students and with an older sample and also when participants were asked to bring to mind expensive purchases, increasing the validity of the proposed mechanism. We discussed the theoretical and applied implications of our results.

### 15.1. Theoretical implications

As discussed in our introduction, experiential purchases have an advantage over their material counterparts in terms of the amount of happiness they are able to elicit. This advantage comes from the fact that experiential purchases satisfy more efficiently the need for relatedness than material purchases (Weingarten and Goodman, 2021). We extended the understanding of type of purchase by showing that experiential purchases satisfied more efficiently the need for autonomy as well. The need for autonomy represents one of the three needs that consumers need to satisfy to experience greater well-being and growth (Ryan and Deci, 2018). Recent developments on the conceptualization of autonomy suggests that it means not only to feel as the origin of decisions and choices, but also make decisions consistent with the true self (Ryan and Deci, 2018). We suggested that consumers reconstructed experiential purchases as more autonomy supportive precisely because they represented better consumers’ true values. We found support for this hypothesis across three studies and additional support in one more study by asking participants explicitly to bring to mind a purchase that truly reflected their core values. These findings provided additional evidence for the importance of self-determination theory as a framework to understand consumers’ motivation. Future studies could assess whether and what type of experiential purchases might also satisfy the need for competence, as suggested before (Weingarten and Goodman, 2021).

Feeling support for autonomy was positively related to greater gratitude across four studies. This result was consistent with a previous investigation showing an experiential advantage in terms of gratitude (Walker et al., 2016). Yet this investigation did not use autonomy support as the mechanism responsible for greater gratitude. Participants in our study felt more grateful for the good things in life when reconstructing experiential than material purchases because they were more consistent with their true values. One previous study found that when consumers wanted to express their true self, they chose experiences over material products (Kim et al., 2016; Bronner and de Hoog, 2018). Our results were consistent with this recent study and showed one affective benefit in the form of gratitude. Future studies could assess consequences of greater gratitude such as repurchase intention, higher sharing in social media, and greater online word of mouth, among others. Gratitude plays an important role in consumer behavior and business scholars should play close attention to it.

In addition, our results showed one consumption benefit, greater ease of justification, as a function of the reconstruction of experiential purchases as more autonomy supportive. To our knowledge, this is the first investigation to examine ease of justification as a potential outcome of type of purchase. Previous studies have separately assessed individual components of ease of justification. For example, one study examined the effect of type of purchase on two different kinds of regret, regret of action and inaction, finding support for the idea experiential purchases were strongly related to regret of inaction (Rosenzweig and Gilovich, 2012). Similarly, another study found that material purchases had more potential for regret than their experiential counterparts (Carter and Gilovich, 2010). Yet, we conceptualized ease of justification as having several indicators, including low levels of regret and guilt, perceptions that the purchase is ease to justify and that it is money well-spent. High levels of ease of justification should be related to positive outcomes such as purchase satisfaction, loyalty, and positive word of mouth, among others (Bui et al., 2011). Future studies could complement our findings by examining additional positive consequences of greater ease of justification coming from experiential purchases.

### 15.2. Applied implications

We see at least three applied implications. First, as the definition of experience implies, experiences are intangible. Yet, marketing scholars recommend to make experiences more tangible by providing consumers with the opportunity to acquire souvenirs, certificates, among others. We suggest that these souvenirs and certificates should be framed around important consumers’ values such as: Authenticity, honesty, living an authentic life, among others. For example, a certificate of completion of a safari to Africa could be framed as: Only for those who dare to have authentic experiences, this diploma certifies you as a participant of meaningful experiences. A second implication is to position brands that provide experiences around core consumers’ values such as authenticity, honesty, and meaningfulness. This type of positioning could be especially relevant for consumers who are seeking self-transcendent experiences. Third, given the developments on artificial intelligence, brands could use this dynamic and interactive technology to remind consumers of previous experiences bought on the web (Chotiyaputta and Zaid, 2022; Shin et al., 2022), facilitating the re-construal of experiential purchases.

### 15.3. Limitations

This investigation had several limitations. First, we used a retrospective design to examine the influence of type of purchase on autonomy support. Future studies could use longitudinal designs to assess how the planning of experiential purchases and the actual consumption of these types of purchases might also support consumers´ autonomy. Second, our experimental manipulation was brief, recalling a purchase and writing about it. It is likely that spontaneous recall of purchases in consumption settings unfolds across time and is shared with others *via* social media and personal conversations. Future studies could use diaries to explore how more spontaneous, natural recall takes place and the positive benefits coming from such recall. Third, a recent meta-analysis (Weingarten and Goodman, 2021) found support for the role of autonomy support. Yet, these authors clearly stated that they did not actually assess autonomy support directly because they just classified different outcomes as autonomy supportive. Our study did assess autonomy support, included other types of purchases, expensive, and examined additional affective and consumption outcome in the form of gratitude and ease of justification. Consequently, the contribution of this article is incremental.

In sum, across four studies, we found support for the proposition that experiential purchases satisfied the need for autonomy more efficiently than material purchases. As a result of this need satisfaction, consumers felt more grateful and had an easier time justifying their purchases. Greater gratitude and ease of justification could potentially lead to important outcomes, which should be explored in future studies.

## Data availability statement

The raw data supporting the conclusions of this article will be made available by the authors, without undue reservation.

## Ethics statement

The studies involving human participants were reviewed and approved by Universidad Anahuac comite de ética. The patients/participants provided their written informed consent to participate in this study.

## Author contributions

RP-D designed the experiments, collected data, analyzed data, and wrote first and final draft. JC-A assisted in designing studies, collected data, read first and final draft. All authors contributed to the article and approved the submitted version.

## Conflict of interest

The authors declare that the research was conducted in the absence of any commercial or financial relationships that could be construed as a potential conflict of interest.

## Publisher’s note

All claims expressed in this article are solely those of the authors and do not necessarily represent those of their affiliated organizations, or those of the publisher, the editors and the reviewers. Any product that may be evaluated in this article, or claim that may be made by its manufacturer, is not guaranteed or endorsed by the publisher.

## Footnotes

^1^Results from this meta-analysis showed an effect size of 0.383. Yet, they estimated that a third was due to publication bias. Hence, we made our estimations with an effect size of 0.26.
